# Prediction of Strain Fatigue Life of HRB400 Steel Based on Meso-Deformation Inhomogeneity

**DOI:** 10.3390/ma13061464

**Published:** 2020-03-23

**Authors:** Lili Jin, Bin Zeng, Damin Lu, Yingjun Gao, Keshi Zhang

**Affiliations:** 1 Key Lab of Disaster Prevent and Structural Safety, Guangxi Key Lab Disaster Prevent and Engineering Safety, College of Civil Engineering and Architecture, Guangxi University, Nanning 530004, China; litiemei666@163.com (L.J.); zengbin379@163.com (B.Z.); luda.min@163.com (D.L.); gaoyj@gxu.edu.cn (Y.G.); 2 Urban Construction and Transportation Engineering Department, Guangxi Polytechnic of Construction, Nanning 530007, China; 3 School of Physical Science and Technology, Guangxi University, Nanning 530004, China; 4 College of Civil and Architectural Engineering, Nanning University, Nanning 530200, China

**Keywords:** strain inhomogeneity, crystal plasticity, BCC, polycrystalline RVE, fatigue indicator parameter, fatigue life prediction

## Abstract

The relationship between strain fatigue life and evolution of meso-deformation inhomogeneity was studied, through the cyclic process of numerical simulation of crystal plasticity compared with the fatigue test of steel hot-rolled ribbed-steel bar 400 (HRB400). The statistical characterization parameters at grain level, including the standard deviation of the dot product of longitudinal stress and strain, the product of the macro stress and the standard deviation of the longitudinal strain, and the product of the macro stress ratio and the standard deviation of the longitudinal strain, were proposed and respectively applied to measure the meso-deformation inhomogeneity of materials. These parameters take the effect of peak stress into account, distinct from the pure strain statistical parameters. The numerical results demonstrate that the low-cycle fatigue life curves of materials are predictable using the new parameters as FIPs (fatigue indicator parameters), and the predictions are more rational than by utilizing the FIPs without considering the peak stress effect.

## 1. Introduction

The issue of metal fatigue has been investigated for a long time, and the prediction of the fatigue life of materials and structures has always been the focus of scholars. In order to evaluate the fatigue life of materials or structures based on existing theories and methods, the fatigue (stress fatigue or strain fatigue) characteristic curve of materials must be measured by a series of experiments [1]. This method was improved and brought to evaluate and predict the fatigue lives of various materials and components by later researchers. Wang et al. presented an experimental study on the fatigue behavior of shot-peened open-hole plates with Q345 steel and evaluated the fatigue life result with an S–N curve (alternating Stress versus Number of cycles to failure) [2]. Ni and Mahadevan developed a strain-based probabilistic fatigue life prediction methodology for spot-welded joints based on p-ε-N curves family and Miner’s rule [3]. In the investigation on multiaxial fatigue damage by Ince and Glinka, their method was also based on the fatigue characteristic curve of material, but more material parameters for multiaxial conditions were needed [4]. Wang et al. researched the microstructure and low-cycle fatigue behavior of Al-9Si-4Cu-0.4Mg-0.3Sc alloy with different casting states and evaluated the fatigue life of this material with the Manson–Coffin formula [5]. All these studies need a series of fatigue experiments to calibrate the parameters in their fatigue life formula.

Scholars also try to seek the parameters reflecting the failure mechanism and damage accumulation of materials, so as to establish a method to evaluate the fatigue life of materials. Ahmadzadeh and Varvani-Farahani studied fatigue damage and life evaluation of SS304 and Al 7050-T7541 alloys by means of energy-based fatigue damage models [6]. Radhakrishnan proposed the relationship between the total fracture energy versus fatigue life, applying medium carbon steel and copper, and a good correlation was obtained between the experimental data and the theoretical prediction [7]. Jiang et al. developed an EVICD (event independent cumulative damage) fatigue prediction model that takes the plastic strain energy as the major contributor to the fatigue damage [8]. Feng et al. established an energy dissipation-based multiaxial fatigue model that allows the fatigue life prediction for a given strain path, and then the proposed model was verified and applied to the AISI 316L stainless steel [9]. Fan et al. proposed a generalized life prediction model on the basis of the hysteresis energy and law of energy conservation for the creep–fatigue interaction [10]. Because the fatigue damage mechanism of materials is still not clear and is difficult to quantitatively describe, the empirical fatigue life formula of materials is used. A series of fatigue experiments are required to calibrate the parameters in these formulas in order to evaluate the fatigue life law of materials. It is a new topic to predict the low cycle fatigue behavior of materials, only according to the known hysteresis behavior and the fatigue life data measured under a single strain amplitude, without fitting a series of test data.

In order to study the process of metal fatigue, it is necessary to consider both the mechanism of slip deformation and the failure of metals at grain level under cyclic loading. A crystal-plasticity cyclic constitutive model for the ratcheting of polycrystalline material considering dislocation substructures was developed by Ren et al. [11]. Farooq et al. studied the complex phenomena of interaction between different grains by a model of crystal plasticity with kinematic hardening under cyclic load [12]. The low-cycle fatigue life prediction of GH4169 superalloy was investigated using the accumulated plastic slip and energy dissipation as fatigue indicator parameter (FIP) [13]. Li et al. presented a multi-scale crystal plasticity model and applied the accumulated slip as FIP to predict crack initiation in precipitate-strengthened steel [14]. Cruzado et al. developed a microstructure-based model that accounts for the effect of grain size, which can be applied to the fatigue simulations and predict the fatigue crack initiation using the local plastic work as an FIP [15,16]. This investigation implied that if the fatigue behavior of a material presents the Coffin–Manson relationship, the value determination of FIP needs two or more fatigue tests with different stress (or strain) amplitude. Lucarini et al. introduce an upscaling approach for micromechanics-based fatigue that can estimate fatigue life at the specimen/component level from the simulation of relatively small representative volume element (RVE) of the polycrystalline microstructure [17]. Liu et al. develop a plasticity model to predict the fatigue crack nucleation of polycrystalline materials in which the accumulated dislocation dipoles are considered to be the origin of damage [18].

Meanwhile, some investigations have been conducted into the possibility for the method of predicting the fatigue life curve of materials. Applying the crystal plastic constitutive model considering the influence of back stress, which can reflect the mechanical behavior of materials under cyclic load, Zhang et al. carried out the numerical simulation with a representative volume element (RVE) containing a certain number of grains [19]. It is found that the hysteretic behavior of FCC (face-centered cubic) polycrystalline copper under symmetric strain can be presented by a numerical simulation. The inhomogeneity of the stress field and the strain field in the RVE can also be reflected by the numerical simulation, and the statistical standard deviation of the longitudinal strain in the RVE can be utilized to measure the inhomogeneity of deformation, which can predict the occurrence of materials’ fatigue failure. Zhang et al. conducted a numerical simulation on the fatigue process of GH4169 and found that the standard deviation of the meso longitudinal strain, the mean value of the first principal strain, and the maximum value of the first principal strain could all be used as characterization parameters for the deformation inhomogeneity [20]. The curves of these parameters with cycle growth can reflect the fatigue damage accumulation of materials, and can be used for the reasonable prediction of the low-cycle fatigue life of materials. On this basis, Zhang et al. further discussed the feasibility of using Shannon differential entropy as an FIP [21]. Liu et al. proposed the FIPs characterizing meso-inhomogeneous deformation on a metal material surface and used them to predict the low-cycle fatigue life [22]. However, these studies all focus on FCC materials. Whether or not the strain inhomogeneity, as the FIPs of materials, is suitable for more general metal, such as steel with body centered cubic (BCC) structure, which is widely used, needs to be further studied.

For this purpose, in this paper, hot-rolled ribbed-steel bar 400 (HRB400) steel is taken as an object to validate the rationality of using strain inhomogeneity as an FIP. Grain of HRB400 has the BCC crystal structure, and its plastic deformation mechanism is mainly slip. By virtue of polycrystalline RVE and crystal plasticity analysis, the numerical simulation of the whole fatigue cyclic process is carried out, and the parameters that can characterize the meso-inhomogeneity of materials are discussed. Three developed FIPs considering the effect of peak stress are proposed, based on the standard deviation of the mesoscopic longitudinal strain and the average mesoscopic first principal strain, and they are the standard deviation of the dot product of mesoscopic stress and strain, the product of the macro stress peak and the standard deviation of the mesoscopic longitudinal strain in RVE, and the product of the macro stress ratio and the standard deviation of the mesoscopic longitudinal strain in RVE. The validity of these parameters in predicting the fatigue life curve of materials is equally verified.

## 2. Material and Strain Fatigue Experiments

**Figure 1:**
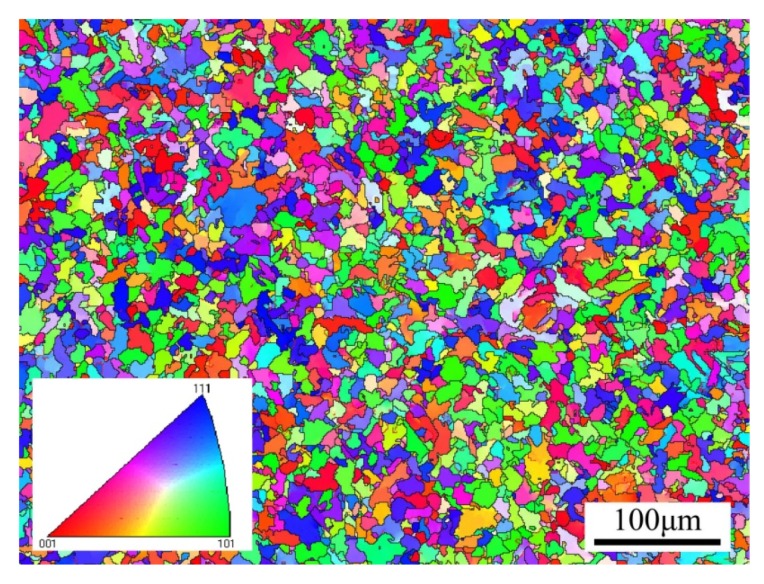
The microstructure of hot-rolled ribbed-steel bar 400 (HRB400) obtained by the EBSD test.

HRB400 (hot-rolled ribbed-steel bar 400) employed in the experiment is a kind of construction steel that is widely used. It is a kind of low carbon steel supplied by Guangxi Guigang Iron and Steel Group Co. LTD (the brand is Guibao). The base material diameter is 25 mm. The density of this material is 7.682 g/cm^3^, and the chemical composition is shown in Table 1. The mechanical property parameters in Table 2 were obtained through the testing of basic mechanical properties (the elongation was measured according to the original gauge length of 30 mm). Figure 1 measured by the EBSD (electron backscatter diffraction) test shows the grain distribution in which boundaries of grains are clearly shown. The average grain size is 15.8 µm, and the different colors in the figure represented the orientation of separate grains.

**Figure 2:**
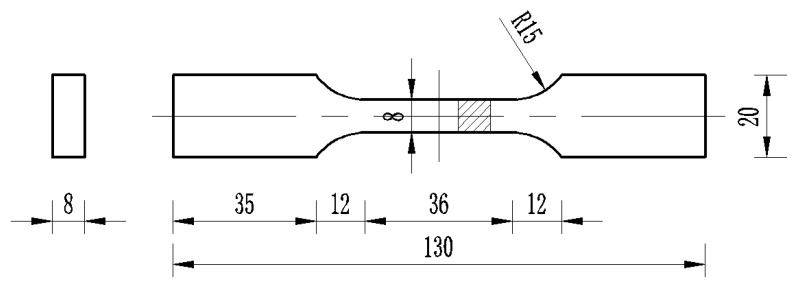
Geometric dimensions of the plate specimen (unit: mm).

The material is processed into the plate sample shown in Figure 2 by wire cutting and surface polishing.

**Figure 3:**
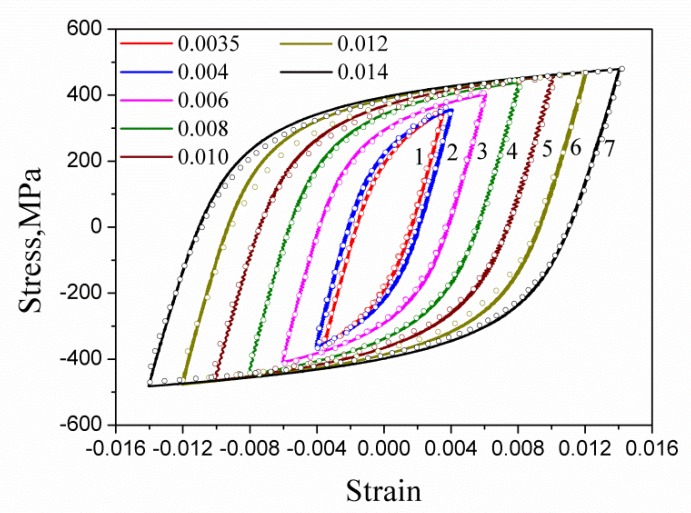
The stable hysteresis loops of tension-compression cyclic test under different strain amplitudes for HRB400.

**Figure 4:**
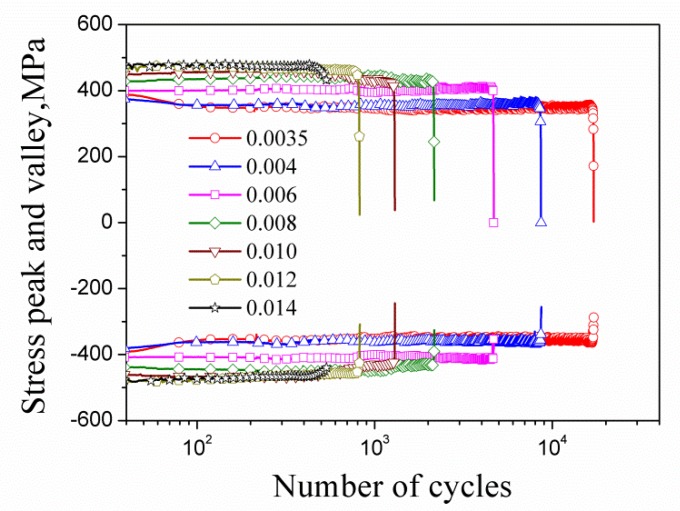
Cyclic stress peak and valley versus the number of cycles at different amplitudes.

The fatigue test was carried out according to ASTM (American Society of Testing Materials) E606 (Standard Practice for Strain-Controlled Fatigue Test). The MTS809 electro-hydraulic servo tension and torsion fatigue testing machine was used, and the gauge distance of the extensometer was 25 mm. The samples were subjected to cyclic symmetrical tension-compression load with constant strain amplitude, and the ambient temperature was room temperature. The loading waveform was a sine wave with a frequency of 0.05 Hz. The strain amplitudes of the test were 0.0035, 0.004, 0.006, 0.008, 0.010, 0.012, and 0.014, respectively. Taking a half-life range, if the error of the peak value of stress is no more than 2% of the mean value of stress amplitude, the cycle in this range is regarded as stabilized; the hysteresis loop is taken at the midpoint of the range. The stable hysteresis loops (solid lines) under different strain amplitudes are shown in Figure 3. The half-life hysteresis loops are also drawn in Figure 3 (plotted with circle points). Figure 4 shows the variation of the stress peak and valley with the number of cycles for the HRB400 at different applied strain amplitudes, which indicates the hysteresis behavior of the steel is basically stabilized.

**Figure 5:**
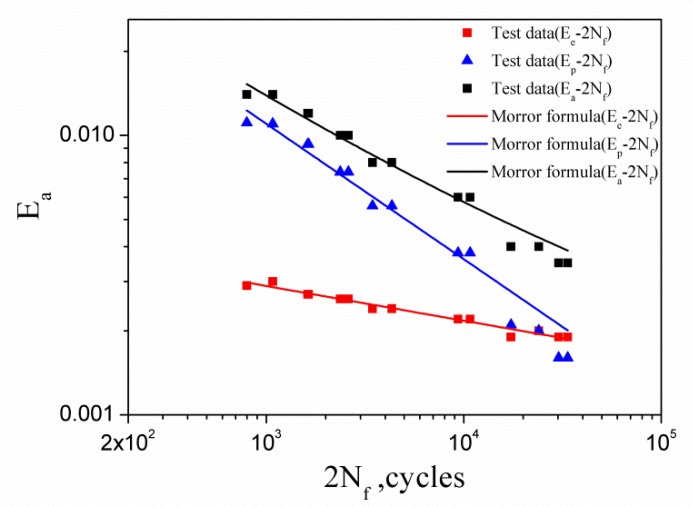
Elastic, plastic, and total strain amplitude versus number of cycles to failure.

For all tests, loading was stopped when the loading obviously dropped and cracks appeared on the sample surface, and the number of cycles corresponding to this time was recorded as the fatigue life of the material, as shown in Table 3. The relationship between the strain amplitudes and the fatigue life in strain fatigue can be represented by the Morrow formula:

(1) $$E_{e}=\frac{\sigma_{f}}{E}{\left(2N_{f}\right)}^{b}$$

(2) $$E_{p}=\varepsilon_{f}{\left(2N_{f}\right)}^{c}$$

(3) $$E_{a}=E_{e}+E_{p}=\frac{\sigma_{f}}{E}{\left(2N_{f}\right)}^{b}+\varepsilon_{f}{\left(2N_{f}\right)}^{c}$$

where $E_{e}$, $E_{p}$, and $E_{a}$ denote the elastic, plastic, and total strain amplitudes, respectively; while $\sigma_{f}$ and b are elastic fatigue constants known as the strength coefficient and exponent, respectively; and $\varepsilon_{f}$ and c are the ductility coefficient and exponent, respectively. The coefficients in these formulas can be obtained by fitting the experimental data in Table 3, which are $\sigma_{f}$ = 1368.01 MPa, *b* = −0.124, $\varepsilon_{f}$ = 0.313, and *c* = −0.485, respectively. The test data and the Morrow curves are all presented in Figure 5.

**Figure 6:**
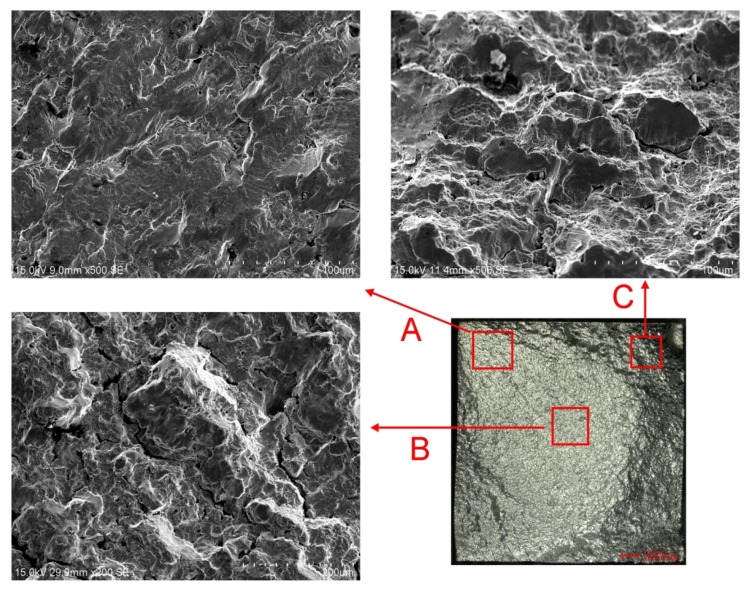
Scanning electron microscope (SEM) micrograph of fracture of specimen applied strain amplitude of 0.004.

The fracture of specimen applied amplitude 0.004 was photographed with an Hitachi s-3400n (Hitachi, Tokyo, Japan) scanning electron microscope (SEM), and is illustrated in Figure 6. The overall appearance of this fracture was observed by a Keyence ultra-depth VHX-2000 microscope (Osaka, Japan). This figure shows the different fracture morphology of the initial fracture area (point A), fracture propagation area (point B), and transient fracture area (point C). In the crack initiation region and propagation region, the fracture shows cleavage characteristics, while the instantaneous fracture region presents typical dimple fracture characteristics.

## 3. The Constitutive Model and the Material Model

### 3.1. The Crystal Plastic Constitutive Model

The Euler velocity gradient tensor L of material points in a grain can be decomposed into the stretching tensor D and the spin rate tensor W according to continuum mechanics. The relationship can be expressed as follows:

(4) $$\begin{array}{l} D=D^{*}+D^{P}=sym\left(L^{*}\right)+sym\left(L^{P}\right),\\ W=W^{*}+W^{p}=asym\left(L^{*}\right)+asym\left(L^{P}\right) \end{array}$$

where $D^{*}$ and $D^{P}$ are the elastic stretching tensor and plastic stretching tensor, respectively; and $W^{*}$ and $W^{p}$ are the elastic spin tensor and the plastic spin tensor, respectively.

In the case of small elastic deformation, the rate constitutive relation can be expressed as the following formula:

(5) $${\dot{\sigma }}^{J}=\overset{\langle 4\rangle }{C}:D^{*}=\overset{\langle 4\rangle }{C}:\left(D-D^{P}\right)$$

where $\overset{<4>}{C}$ is the fourth-order elastic constitutive tensor of the crystal in a global coordinate system fixed during calculations. The crystal axis coordinate system of each crystal is calculated according to its initial orientation and rotation history during deformation. ${\dot{\sigma }}^{J}$ is the Jaumann rate of Cauchy stress, in which the effect of rigid body rotation during deformation has been deducted, and can be calculated as follows:

(6) $${\dot{\sigma }}^{J}=\dot{\sigma }-W\cdot \sigma +\sigma \cdot W$$

where σ and $\dot{\sigma }$ are the Cauchy stress and its rate, respectively. The logarithmic strain increment Δε can be obtained by integral of stretching tensor D for the time increment period of t and t+Δt. Therefore, the cumulative incremental change of σ can be updated as follows by using Equations (5) and (6):

(7) σt+Δt−σ|tt+Δt=ΔσJorσt+Δt=σ|tt+Δt+C<4>:(Δε−Δεp)

where $\Delta \varepsilon^{p}$ is the logarithmic plastic strain increment, and σ|tt+Δt is σ|t under the corresponding configuration at t+Δt.

For the plastic deformation under slipping mechanism, based on the evolution law of slip shear strain of single crystal proposed by Hutchinson [23], the relationship between shear stress and shear strain rate in the α-slip system can be described referring to the Chaboche model to consider the influence of Bauschinger effect by introducing the back stress into it [24,25].

(8) $${\dot{\gamma }}^{\left(\alpha \right)}={\dot{\gamma }}_{0}\mathrm{sgn}\left(\tau^{\left(\alpha \right)}-x^{\left(\alpha \right)}\right){\left|\frac{\tau^{\left(\alpha \right)}-x^{\left(\alpha \right)}}{g^{\left(\alpha \right)}}\right|}^{k}$$

where $\tau^{\left(\alpha \right)}$ and $x^{\left(\alpha \right)}$ are the shear stress and back stress on the α-slip system, respectively; $g^{\left(\alpha \right)}$ defines the scalar function describing the elastic domain of the shear stress for the α-slip system; ${\dot{\gamma }}_{0}$ is a constant denoting the reference shear strain rate; and *k* denotes the rate sensitivity parameter. The evolution of back-stress $x^{\left(\alpha \right)}$ is introduced as follows [26]:

(9) $${\dot{x}}^{\left(\alpha \right)}=a{\dot{\gamma }}^{\left(\alpha \right)}-c\left[1-e_{1}\left(1-\exp\left(-e_{2}\gamma \right)\right)\right]x^{\left(\alpha \right)}\left|{\dot{\gamma }}^{\left(\alpha \right)}\right|-px^{\left(\alpha \right)}$$

where parameter *a* is the material constant describing the linear hardening of slip systems, parameters *c* and *p* are material constants representing nonlinear hardening characteristics, and $e_{1}$ and $e_{2}$ are material constants that reflect the law of cyclic hardening or softening. The identification of these four parameters is based on cyclic tests combined with numerical simulation.

The evolution of scalar function $g^{\left(\alpha \right)}$ is calculated according to the formula suggested by Pan and Rice [27]:

(10) $${\dot{g}}^{\left(\alpha \right)}\left(\gamma \right)=\sum_{\beta =1}^{n}h_{\alpha \beta }\left(\gamma \right)\left|{\dot{\gamma }}^{\left(\beta \right)}\right|,\gamma =\int \sum_{\beta =1}^{n}\left|d\gamma^{\left(\beta \right)}\right|$$

where $h_{\alpha \beta }$ is the slip-plan hardening modulus. Furthermore, Hutchinson proposed that the modulus can be calculated as follows [28]:

(11) $$h_{\alpha \beta }\left(\gamma \right)=h\left(\gamma \right)\left[q+\left(1-q\right)\delta_{\alpha \beta }\right]$$

where *q* is a constant and h(γ) is given according to Chang and Asaro [29] as follows:

(12) $$h\left(\gamma \right)=h_{0}{\mathrm{sech}}^{2}\left(\frac{h_{0}\gamma }{\tau_{s}-\tau_{0}}\right)$$

where $h_{0}$ is the initial hardening rate; and $\tau_{0}$ and $\tau_{s}$ are the critical resolved shear stress and the saturation value, respectively. These parameters are regarded as material constants that are obtained by experiments and numerical simulation.

For the α-slip system, $m^{\left(\alpha \right)}$ and $n^{\left(\alpha \right)}$ are denoted as the unit vectors of initial slip direction and the unit normal vector of the initial slip plane, respectively. Referring to the methods, the Schmid tensor, which reflects the relationship between shear stress–strain of slip system and stress–strain under global rectangular coordinate system, can be written as follows [30,31]:

(13) $$P^{\left(\alpha \right)*}=\frac{1}{2}\left(m^{\left(\alpha \right)*}n^{\left(\alpha \right)*}+n^{\left(\alpha \right)*}m^{\left(\alpha \right)*}\right),\;\;\mathrm{and}\;m^{\left(\alpha \right)*}=F^{*}\cdot m^{\left(\alpha \right)},\;n^{\left(\alpha \right)*}=n^{\left(\alpha \right)}\cdot F^{*-1}$$

where $F^{*}$ is the elastic part of the deformation gradient tensor. The increment of logarithmic strain tensor in Equation (7) is calculated as follows [32]:

(14) $$\Delta \varepsilon^{p}=\sum_{\alpha =1}^{n}P^{\left(\alpha \right)*}\Delta \gamma^{\left(\alpha \right)}$$

where $\Delta \gamma^{\left(\alpha \right)}$ is obtained by the integral of ${\dot{\gamma }}^{\left(\alpha \right)}$. According to the Schmid law, we have the following:

(15) $$\tau^{\left(\alpha \right)}=P^{\left(\alpha \right)*}:\sigma$$

The calculation process and algorithm of the above model are detailed in [19,26], which can be implemented by the user material subroutine UMAT of ABAQUS (Dassault Systemes, Paris, France).

### 3.2. Polycrystalline RVE Material Model

**Figure 7:**
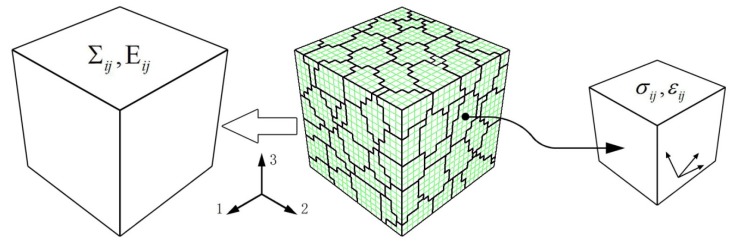
The representative volume element (RVE) with 8000 elements.

The RVE material model, containing 8000 elements and shown in Figure 7, was used for numerical simulation. Generally speaking, the more elements the material model contains, the higher the calculation accuracy will be. However, an appropriate reduction of the elements in the RVE can substantially reduce the time consumption, while the effect of the reduction of elements on the calculation results is still acceptable [21].

On the positive surfaces orthogonal to the three-axis, the displacement along normal is specified; on the negative surfaces orthogonal to the 1-axis, 2-axis, and 3-axis, are fixed in the normal direction; on the positive surfaces perpendicular to the one-axis and two-axis, the macro normal stress and shear stress are set as zero, respectively, while the surfaces are kept as plane. Under such a boundary condition, the state of the RVE is nearly the same as a material unit on the working section of the samples under symmetric tension–compression cyclic load controlled by strain. The six surfaces of the RVE will always remain plane (macroscopic homogeneous deformation), and the RVE maintains under macroscopic uniaxial stress state during the deformation process. As principle analysis, the effects of metallurgical defects, grain boundary effects, and size effects are not considered in the calculation.

The macroscopic mechanical behavior of metal can be reflected by the statistical mean values of stress and strain in the RVE, and the mesoscopic mechanical behavior of inhomogeneity of materials can be reflected by the statistical standard deviations of stress and strain using the above method. The statistical mean and standard deviation of stress and strain are calculated as follows:

(16) $$\begin{array}{c} {\bar{\sigma }}_{ij}=\sum_{k=1}^{n_{RVE}}{\left(\sigma_{ij}\right)}_{k}p_{k}\;,{\bar{\varepsilon }}_{ij}=\sum_{k=1}^{n_{RVE}}{\left(\varepsilon_{ij}\right)}_{k}p_{k}\\ {\hat{\sigma }}_{ij}=\sqrt{\sum_{k=1}^{n_{RVE}}{\left(\sigma_{ij}\right)}_{k}^{2}p_{k}-{\bar{\sigma }}_{ij}^{2}}\;,\;{\hat{\varepsilon }}_{ij}=\sqrt{\sum_{k=1}^{n_{RVE}}{\left(\varepsilon_{ij}\right)}_{k}^{2}p_{k}-{\bar{\varepsilon }}_{ij}^{2}} \end{array}$$

where $n_{RVE}$ is the amount of the elements in the RVE; ${\left(\sigma_{ij}\right)}_{k}$ and ${\left(\varepsilon_{ij}\right)}_{k}$ are the mesoscopic Cauchy stress tensor components and logarithmic strain tensor, respectively; the subscript k is the sequence number of the finite element; and $p_{k}=\Delta V_{k}/V$, where $\Delta V_{k}$ denotes the volume of the kth element and V the total volume of the RVE.

### 3.3. Model Parameters of Crystal Plastic Constitutive Model

**Figure 8:**
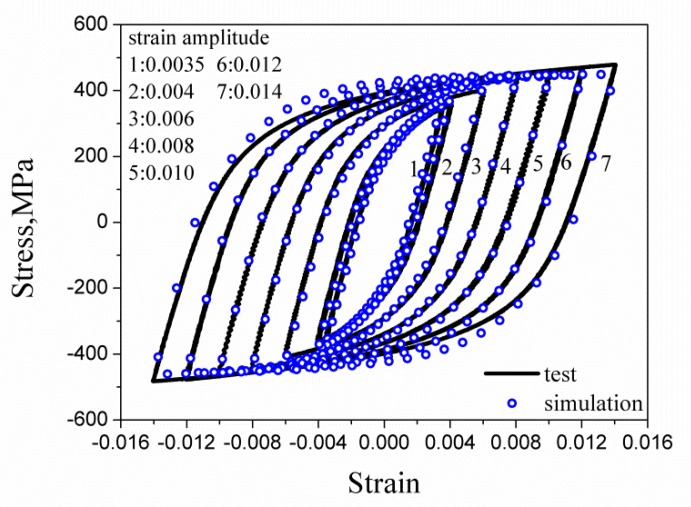
The comparison of experiment and simulation of stable hysteresis loops of the symmetric strain cycle at different strain amplitudes.

The parameters of the crystal plastic constitutive model for HRB400 shown in Table 4 were calibrated by the trial-and-error method. Figure 8 shows the comparison between the simulated stable hysteretic curve and the experimental curve. The conclusion can be drawn from Figure 8 that the numerical simulation can reasonably reproduce the macroscopic mechanical behavior of the material by the crystal plastic model combined with the RVE.

## 4. Low-Cycle Fatigue Life Prediction Based on Inhomogeneity of Material Deformation

### 4.1. The Indicator Parameters of Material Deformation Inhomogeneity

Not only can the macroscopic cyclic mechanical behavior of the material be simulated, but also the parameters that describe the inhomogeneity of the internal deformation of the material can be calculated from the simulation, because the differences in grain size, shape, and orientation were taken into account while the RVE was constructed. These two parameters that can measure the inhomogeneity are the standard deviation of the mesoscopic longitudinal strain ${\hat{\varepsilon }}_{l\underline{l}}$ and the average mesoscopic first principal strain ${\bar{\varepsilon }}_{1}$. Their numerical values are different, but their evolution laws are approximately the same. They can be used as the FIPs to apply to the prediction of material fatigue life in the case of symmetrical strain cycle, according to the studies in the literature [19,20]. The fatigue failure criteria can be expressed by the following:

(17) $${\hat{\varepsilon }}_{l\underline{l}}={\hat{\varepsilon }}_{l\underline{l}fatigue}$$

(18) $${\bar{\varepsilon }}_{1}={\bar{\varepsilon }}_{1fatigue}$$

### 4.2. The Parameters of Deformation Inhomogeneity Considering the Influence of Peak Stress

The fatigue failure of materials is also related to the magnitude of stress in addition to the evolution of meso-deformation inhomogeneity. On the basis of this consideration, three FIPs including the influence of peak stress are proposed in this paper.

Firstly, ${\hat{\varepsilon }}_{l\underline{l}}^{I}$, which is the statistical standard deviation of the dot product of mesoscopic strain $\varepsilon_{l\underline{l}}$, and stress $\sigma_{l\underline{l}}$ in the RVE at the tension peak can be expressed as follows (refer to Equation (16)):

(19) $${\hat{\varepsilon }}_{l\underline{l}}^{I}=\sqrt{\sum_{k=1}^{n_{RVE}}{\left(\varepsilon_{l\underline{l}}^{I}\right)}_{k}^{2}p_{k}-{\bar{\varepsilon }}_{l\underline{l}}^{I}{}^{2}}\;\varepsilon_{l\underline{l}}^{I}=\varepsilon_{l\underline{l}}\cdot \sigma_{l\underline{l}}\;(\mathrm{do}\;\mathrm{not}\;\mathrm{sum})$$

Secondly, the stress peak of the macroscopic stability hysteretic curve is taken as the weighted coefficient of the standard deviation of the mesoscopic longitudinal strain ${\hat{\varepsilon }}_{l\underline{l}}$, and ${\hat{\varepsilon }}_{l\underline{l}}^{\mathrm{II}}$ can be achieved. ${\hat{\varepsilon }}_{l\underline{l}}^{\mathrm{II}}$ can be calculated as follows:

(20) $${\hat{\varepsilon }}_{l\underline{l}}^{\mathrm{II}}=\sum_{\max}\cdot {\hat{\varepsilon }}_{l\underline{l}}$$

**Figure 9:**
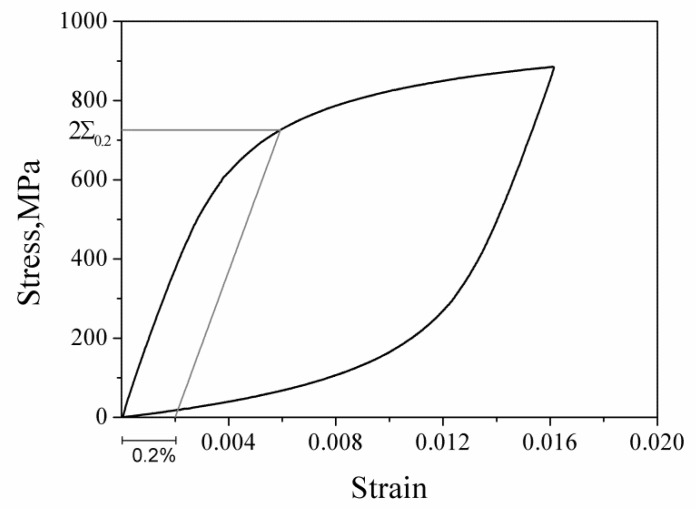
The definition of macroscopic yield stress.

Thirdly, ${\hat{\varepsilon }}_{l\underline{l}}^{\mathrm{III}}$ can be obtained by taking the ratio of stress peak to macroscopic yield stress as the weighted coefficient of ${\hat{\varepsilon }}_{l\underline{l}}$. ${\hat{\varepsilon }}_{l\underline{l}}^{\mathrm{III}}$ can be calculated by the following:

(21) $${\hat{\varepsilon }}_{l\underline{l}}^{\mathrm{III}}=\frac{\sum_{\max}}{\sum_{0.2}}\cdot {\hat{\varepsilon }}_{l\underline{l}}$$

where $\sum_{0.2}$ is the macroscopic yield stress on the stable hysteresis state, and its value is determined in light terms of the elastic range of the test hysteresis loops (cf. Figure 3) and the offset residual plastic strain of 0.2%, as shown in Figure 9. It is necessary to point out that the value of $\sum_{0.2}$ is slightly different depending on the strain amplitude.

If these parameters, ${\hat{\varepsilon }}_{l\underline{l}}^{I}$, ${\hat{\varepsilon }}_{l\underline{l}}^{\mathrm{II}}$, and ${\hat{\varepsilon }}_{l\underline{l}}^{\mathrm{III}}$ are utilized as FIPs, we must check whether they can give a reasonable judgment on the occurrence of material fatigue failure according to the corresponding critical value that is reached, that is,

(22) $${\hat{\varepsilon }}_{l\underline{l}}^{I}={\hat{\varepsilon }}_{l\underline{l}fatigue}^{I}$$

(23) $${\hat{\varepsilon }}_{l\underline{l}}^{\mathrm{II}}={\hat{\varepsilon }}_{l\underline{l}fatigue}^{\mathrm{II}}$$

(24) $${\hat{\varepsilon }}_{l\underline{l}}^{\mathrm{III}}={\hat{\varepsilon }}_{l\underline{l}fatigue}^{\mathrm{III}}$$

where ${\hat{\varepsilon }}_{l\underline{l}fatigue}^{I}$, ${\hat{\varepsilon }}_{l\underline{l}fatigue}^{\mathrm{II}}$, and ${\hat{\varepsilon }}_{l\underline{l}fatigue}^{\mathrm{III}}$ are the critical values of ${\hat{\varepsilon }}_{l\underline{l}}^{I}$, ${\hat{\varepsilon }}_{l\underline{l}}^{\mathrm{II}}$, and ${\hat{\varepsilon }}_{l\underline{l}}^{\mathrm{III}}$, respectively.

### 4.3. Prediction of Low Cycle Fatigue Life of Materials

**Figure 10:**
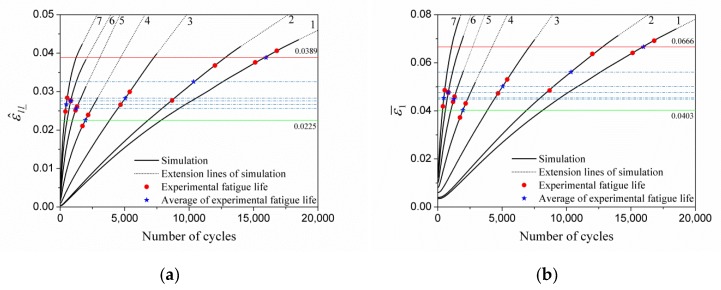

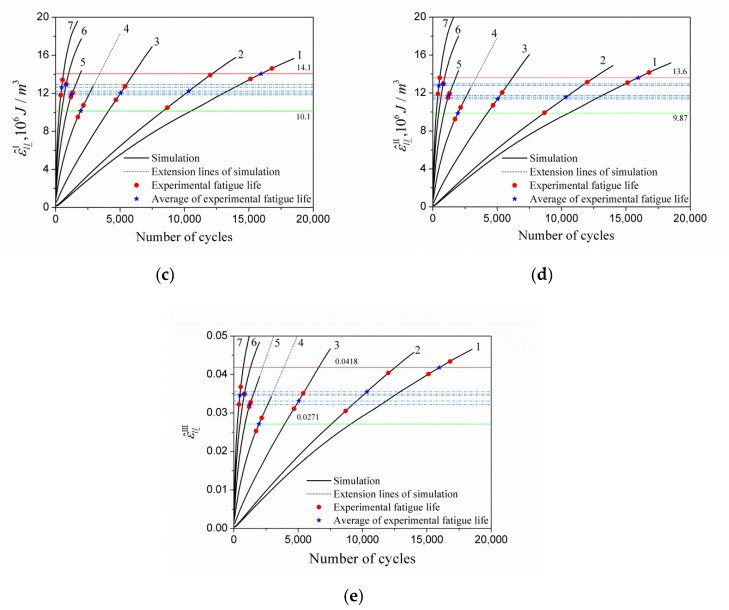
The evolution of various fatigue indicator parameters (FIPs) under different strain amplitudes at the tension peak: (**a**) ${\hat{\varepsilon }}_{l\underline{l}}$; (**b**) ${\bar{\varepsilon }}_{1}$; (**c**) ${\hat{\varepsilon }}_{l\underline{l}}^{I}$; (**d**) ${\hat{\varepsilon }}_{l\underline{l}}^{\mathrm{II}}$; and (**e**) ${\hat{\varepsilon }}_{l\underline{l}}^{\mathrm{III}}$. The horizontal lines in the figures represent respective critical values of the FIPs in Table 5, and the red line indicates the maximum value and the green minimum.

Figure 10 shows the evolution curves of these five indicator parameters characterizing the meso-inhomogeneous deformation of materials with the number cycles. It can be concluded that ${\hat{\varepsilon }}_{l\underline{l}}^{I}$, ${\hat{\varepsilon }}_{l\underline{l}}^{\mathrm{II}}$, ${\hat{\varepsilon }}_{l\underline{l}}^{\mathrm{III}}$, ${\hat{\varepsilon }}_{l\underline{l}}$, and ${\bar{\varepsilon }}_{1}$ all increase with the number of cycles, and the evolution law is similar. The test fatigue lives of materials under different strain amplitudes are marked and the relationship between lives and indicator parameters is shown in Figure 10. The later stage of the evolution curve shows an approximate linear growth according to the calculation results of this paper and in the literature [19,20]. Linear extension line (black dot line) is employed in the figure after the test fatigue life is exceeded in order to reduce time consumption. The abscissa of red solid dot on the curve of any specified strain amplitude cycle corresponds to the fatigue life of a single specimen, and the abscissa of blue pentagram corresponds to the average life of all specimens. The ordinate of these marked dots corresponds to the values of FIPs, which are shown in Table 5.

The vertical coordinate value of the blue star point of the corresponding curve in Figure 10 is regarded as the approximate critical value of the corresponding parameter. A horizontal line can be drawn through the point. The intersected point of the horizontal line and the parameter curve of other strain amplitude cycles by simulation is the predicted life of the corresponding strain amplitude cycle. Therefore, according to the fatigue test of any strain amplitude, the fatigue life of the cyclic loaded specimen at other strain amplitudes can be predicted through Figure 10. The prediction of fatigue life based on parameters ${\hat{\varepsilon }}_{l\underline{l}}$, ${\bar{\varepsilon }}_{1}$, ${\hat{\varepsilon }}_{l\underline{l}}^{I}$, ${\hat{\varepsilon }}_{l\underline{l}}^{\mathrm{II}}$, and ${\hat{\varepsilon }}_{l\underline{l}}^{\mathrm{III}}$ is listed in Table 6, Table 7, Table 8, Table 9 and Table 10.

**Figure 11:**
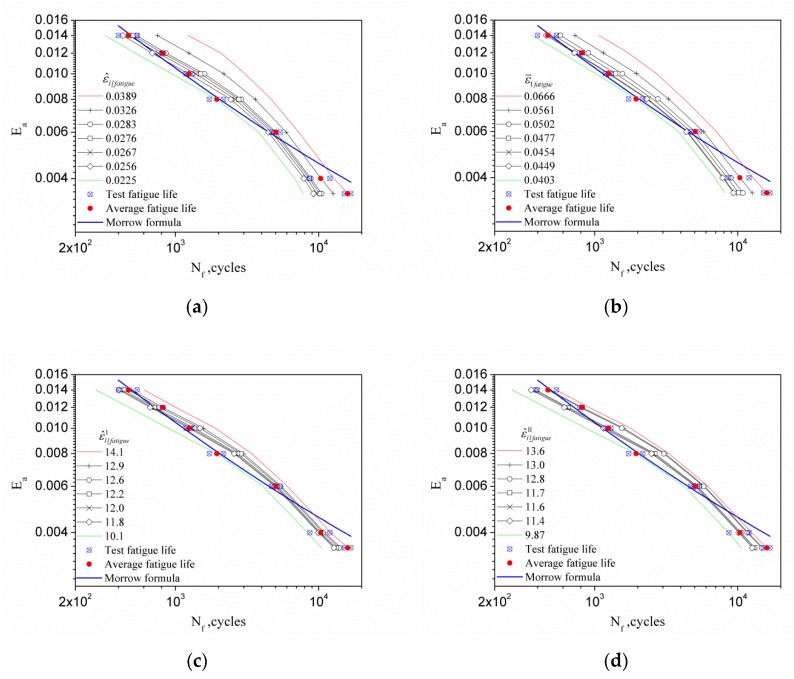

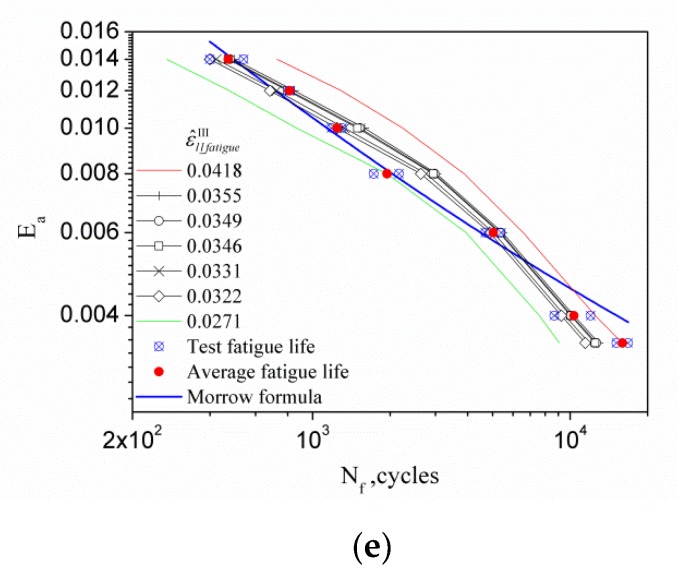
The predicted fatigue life curves $E_{a}∼N_{f}$ based on various limit value of FIPs: (**a**) ${\hat{\varepsilon }}_{l\underline{l}fatigue}$; (**b**) ${\hat{\varepsilon }}_{1fatigue}$; (**c**) ${\hat{\varepsilon }}_{l\underline{l}fatigue}^{I}$; (**d**) ${\hat{\varepsilon }}_{l\underline{l}fatigue}^{\mathrm{II}}$; and (**e**) ${\hat{\varepsilon }}_{l\underline{l}fatigue}^{\mathrm{III}}$.

A predicted fatigue life curve can be drawn based on the fatigue test of any strain amplitude according to Table 6, Table 7, Table 8, Table 9 and Table 10. Figure 11 shows the prediction of fatigue life curves obtained from various strain amplitude fatigue tests and FIPs, respectively, and the Morrow curve obtained by fitting all test data. It is obvious that the introduction of inhomogeneous deformation indicator parameters affected by peak stress is reasonable from the predicted fatigue life curve shown in the figure. It is necessary to point out that each prediction only employed the test data obtained at a single strain amplitude, whereas the Morrow curve must use all test data.

### 4.4. Error Test of Life Prediction

**Figure 12:**
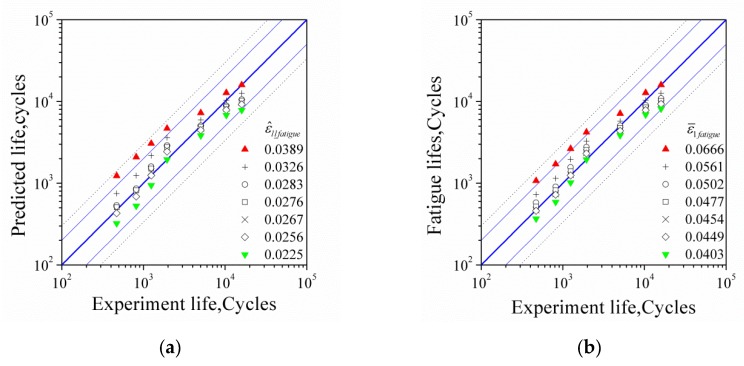

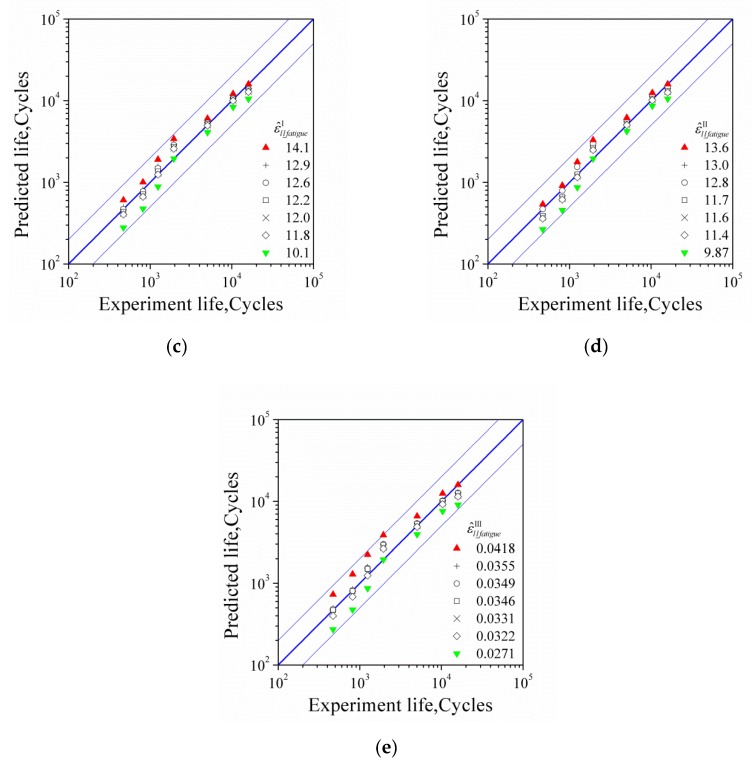
Assessment of the error between the predicted and test fatigue lives with different FIPs: (**a**) ${\hat{\varepsilon }}_{l\underline{l}fatigue}$; (**b**) ${\hat{\varepsilon }}_{1fatigue}$; (**c**) ${\hat{\varepsilon }}_{l\underline{l}fatigue}^{I}$; (**d**) ${\hat{\varepsilon }}_{l\underline{l}fatigue}^{\mathrm{II}}$; and (**e**) ${\hat{\varepsilon }}_{l\underline{l}fatigue}^{\mathrm{III}}$.

In order to check the error between predicted and test lives, Figure 12 was plotted. In the figure, the test and predicted values of fatigue lives of cyclic experiments were the abscissa and ordinate, respectively. The validities of these five FIPs, ${\hat{\varepsilon }}_{l\underline{l}fatigue}$, ${\hat{\varepsilon }}_{1fatigue}$, ${\hat{\varepsilon }}_{l\underline{l}fatigue}^{I}$, ${\hat{\varepsilon }}_{l\underline{l}fatigue}^{\mathrm{II}}$, and ${\hat{\varepsilon }}_{l\underline{l}fatigue}^{\mathrm{III}}$, in prediction of fatigue life were compared. The predicted results with the maximum and minimum values of FIPs are also shown in this figure. The blue thick solid line represents the ideal prediction result, that is, the predicted life is completely consistent with the experimental life. The blue thin solid line indicates the two times error band, and the black dotted line indicates the three times error band. Except that the result predicted by the maximum value of ${\hat{\varepsilon }}_{l\underline{l}fatigue}$ and ${\hat{\varepsilon }}_{1fatigue}$ is close to 2.5 times error (the critical value of the indicator parameter was determined by the cyclic test with a strain amplitude of 0.35%), the error range of other predictions is within 2 times. It should be pointed out that it is more reasonable to judge the fatigue failure with the new suggested FIPs, indicating that the tensile stress at the peak of the cycle is an important factor affecting the initiation of the fatigue crack.

## 5. Conclusions

The research presented in this paper leads to the following conclusions:

(1) The fatigue life prediction method based on the parameter of meso-deformation inhomogeneity is also applicable for BCC materials.
(2) Considering the influence of the stress peak, three improved FIPs are suggested on the basis of the indicator parameter ${\hat{\varepsilon }}_{l\underline{l}}$. These new parameters are similar to each other in describing the evolution of the inhomogeneity of the meso-deformation along with the cycle.
(3) More reasonable fatigue life prediction results can be obtained using the FIPs proposed in this paper, which means that the description of fatigue damage accumulation by the new indicator parameters is closer to the actual process.

## Figures and Tables

**Table 1:** Chemical composition of hot-rolled ribbed-steel bar 400 (HRB400) (mass fraction %).

C	Si	Mn	P	S	Ceq	Fe
0.23	0.37	1.30	0.032	0.015	0.48	rest

**Table 2:** Mechanical properties of HRB400.

E/MPa	G/MPa	μ	$\sigma_{0}/\mathrm{MPa}$	$\sigma_{b}/\mathrm{MPa}$	δ/%	ψ/%	${\bar{\varepsilon }}_{f}$
201,000	75,100	0.34	410	615	33.7	58.2	0.871

**Table 3:** Fatigue life of HRB400 under different strain amplitudes.

$E_{a}$	0.0035	0.004	0.006	0.008	0.010	0.012	0.014
$N_{f}$	16,800/15,128	8681/12,009	4680/5392	2165/1727	1185/1302	810/820	399/540
${\bar{N}}_{f}$	15964	10341	5036	1946	1244	815	470

**Table 4:** Crystal plastic model parameters for HRB400.

Elastic Constants			Material Parameters of the Crystal Viscoplastic Model										
$C_{11}$ GPa	$C_{12}$ GPa	$C_{44}$ GPa	$\tau_{0}$ MPa	$\tau_{s}$ MPa	$h_{0}$ MPa	a GPa	c GPa	*p* $s^{-1}$	$e_{1}$	$e_{2}$	${\dot{\gamma }}_{0\;}\;s^{-1}$	*q*	*k*
293.4	158.0	67.6	103	109	40	31	0.42	0	0	0	0.001	1	200

**Table 5:** The values of fatigue indicator parameters (FIPs) corresponding to the test fatigue life of HRB400.

E_a_	Test Life (Average)	${\hat{\varepsilon }}_{l\underline{l}fatigue}$	${\bar{\varepsilon }}_{1fatigue}$	${\hat{\varepsilon }}_{l\underline{l}fatigue}^{I}$	${\hat{\varepsilon }}_{l\underline{l}fatigue}^{\mathrm{II}}$	${\hat{\varepsilon }}_{l\underline{l}fatigue}^{\mathrm{III}}$
0.0035	15964	0.0389	0.0666	14.1	13.6	0.0418
0.004	10341	0.0326	0.0561	12.2	11.6	0.0355
0.006	5036	0.0283	0.0502	12.0	11.4	0.0331
0.008	1946	0.0225	0.0403	10.1	9.87	0.0271
0.010	1244	0.0256	0.0449	11.8	11.7	0.0322
0.012	815	0.0276	0.0477	12.9	13.0	0.0349
0.014	470	0.0267	0.0454	12.6	12.8	0.0346

**Table 6:** The prediction of fatigue lives applying various critical values of ${\hat{\varepsilon }}_{l\underline{l}fatigue}$ in the representative volume element (RVE).

${\hat{\varepsilon }}_{l\underline{l}fatigue}$	0.0389 E_a_ = 0.0035	0.0326 E_a_ = 0.004	0.0283 E_a_ = 0.0.006	0.0225 E_a_ = 0.008	0.0256 E_a_ = 0.010	0.0276 E_a_ = 0.012	0.0267 E_a_ = 0.014	Test Life (Average)
$E_{a}$	$N_{f}$							
0.0035	15,964	12,611	10,526	7819	9210	10,187	9732	15,964
0.004	12,800	10,341	8844	6807	7892	8609	8286	10,341
0.006	7262	5939	5036	3822	4466	4894	4704	5036
0.008	4681	3615	2885	1946	2438	2772	2619	1946
0.010	3061	2176	1597	945	1244	1506	1385	1244
0.012	2083	1241	858	525	687	815	759	815
0.014	1230	748	534	322	429	506	470	470

**Table 7:** The prediction of fatigue lives applying various critical values of ${\bar{\varepsilon }}_{1fatigue}$ in the RVE.

${\bar{\varepsilon }}_{1fatigue}$	0.0666 E_a_ = 0.0035	0.0561 E_a_ = 0.004	0.0502 E_a_ = 0.006	0.0403 E_a_ = 0.008	0.0449 E_a_ = 0.010	0.0477 E_a_ = 0.012	0.0454 E_a_ = 0.014	Test life (Average)
$E_{a}$	$N_{f}$							
0.0035	15,964	12,625	10,873	8148	9344	10,127	9480	15,964
0.004	12,802	10,341	8988	6898	7842	8443	7950	10,341
0.006	7109	5769	5036	3849	4382	4725	4444	5036
0.008	4212	3281	2765	1946	2312	2545	2354	1946
0.010	2699	1961	1562	1016	1244	1404	1271	1244
0.012	1712	1147	904	587	723	815	739	815
0.014	1071	728	577	367	458	518	470	470

**Table 8:** The prediction of fatigue lives applying various critical values of ${\hat{\varepsilon }}_{l\underline{l}fatigue}^{I}$ in the RVE.

${\hat{\varepsilon }}_{l\underline{l}fatigue}^{I}$	14.1 E_a_ = 0.0035	12.2 E_a_ = 0.004	12.0 E_a_ = 0.006	10.1 E_a_ = 0.008	11.8 E_a_ = 0.010	12.9 E_a_ = 0.012	12.6 E_a_ = 0.014	Test Life (Average)
$E_{a}$	$N_{f}$							
0.0035	15,964	13,345	13,031	10,514	12,801	14,322	13,931	15,964
0.004	12,147	10,341	10,133	8345	9974	11,005	10,733	10,341
0.006	6049	5147	5036	4094	4951	5491	5353	5036
0.008	3411	2718	2635	1946	2572	2981	2874	1946
0.010	1903	1356	1292	885	1244	1569	1483	1244
0.012	1005	714	687	475	665	815	772	815
0.014	606	436	418	279	404	494	470	470

**Table 9:** The prediction of fatigue lives applying various critical values of ${\hat{\varepsilon }}_{l\underline{l}fatigue}^{\mathrm{II}}$ in the RVE.

${\hat{\varepsilon }}_{l\underline{l}fatigue}^{\mathrm{II}}$	13.6 E_a_ = 0.0035	11.6 E_a_ = 0.004	11.4 E_a_ = 0.006	9.87 E_a_ = 0.008	11.7 E_a_ = 0.010	13.0 E_a_ = 0.012	12.8 E_a_ = 0.014	Test Life (Average)
$E_{a}$	$N_{f}$							
0.0035	15,964	12,886	12,617	10,542	13,151	14,944	14,699	15,964
0.004	12,514	10,341	10,152	8633	10,527	11,805	11,627	10,341
0.006	6189	5137	5036	4235	5235	5864	5778	5036
0.008	3303	2565	2493	1946	2635	3109	3043	1946
0.010	1777	1197	1152	867	1244	1597	1549	1244
0.012	913	635	613	456	656	815	792	815
0.014	540	374	360	265	387	483	470	470

**Table 10:** The prediction of fatigue lives applying various critical values of ${\hat{\varepsilon }}_{l\underline{l}fatigue}^{\mathrm{III}}$ in the RVE.

${\hat{\varepsilon }}_{l\underline{l}fatigue}^{\mathrm{III}}$	0.0418 E_a_ = 0.0035	0.0355 E_a_ = 0.004	0.0331 E_a_ = 0.006	0.0271 E_a_ = 0.008	0.0322 E_a_ = 0.010	0.0349 E_a_ = 0.012	0.0346 E_a_ = 0.014	Test Life (Average)
$E_{a}$	$N_{f}$							
0.0035	15,964	12,912	11,845	9084	11,447	12,635	12,499	15,964
0.004	12,520	10,341	9542	7580	9241	10,140	10,042	10,341
0.006	6584	5470	5036	3945	4876	5363	5310	5036
0.008	3874	3033	2762	1946	2634	2979	2940	1946
0.010	2242	1591	1331	867	1244	1527	1495	1244
0.012	1291	849	725	475	683	815	800	815
0.014	728	496	423	272	399	478	470	470
